# Registration and the Ministry of Health

**Published:** 1919-06-28

**Authors:** 


### THE MATRONS' AND SISTERS' DEPARTMENT.

### REGISTRATION AND THE MINISTRY OF HEALTH.

In his remarkable speech at the fourth annual meeting of the College of Nursing held at Manchester on June 18, Sir Arthur Stanley stated that since both Houses of Parliament had affirmed the principle of State Registration for Nurses, obviously the best solution would be for the Government itself to take the matter in hand. The fact that the Ministry of Health is now established facilitates this step. The College of Nursing would welcome it, and we have no reason to suppose that the Central Committee would be other than satisfied that the Government should make the registration of nurses their own measure.

There is no special magic about Government which confers immunity from mistakes upon the measures they undertake. But undoubtedly Bills which Government takes in hand are secured from certain dangers which may lurk in those promoted by private persons. In the first place, State registration, if dealt with by the Ministry of Health, would be removed once and for all from the sphere of any who could be suspected of having an axe of their own to grind. There is a cynical strand in men's minds which tempts them to inquire, when confronted by energy and enthusiasm in a cause, however good, "What do these enthusiasts stand to gain by it personally?" And when, as in the present instance, there are strong differences of opinion as to the form legislation should take, existing side by side with substantial agreement about the principle on which it should be based, there is a good case for invoking an impartial authority, not merely to decide between two opposite opinions, as Parliament must do, but to build a thoroughly satisfactory measure both sides would accept.

It must be remembered that the registration of nurses by the State, simple as it sounds in theory, is an extremely difficult and even contentious matter in practice. It closely affects the interests of all classes of institutions. It is, moreover, a national measure. The proper regulation of the nursing service is a matter which affects every one, rich and poor, not merely nurses themselves. It is a matter of the most acute concern to the Ministry of Health, which must sooner or later be compelled to deal with it, even if the present proposals for legislation prove abortive. A large number of nurses are actually in Government employ, or working under departments which will assuredly soon be linked up under the Ministry of Health. It is an invidious matter for private Bills to legislate for nurses employed under the Local Government Board, for instance, or in the Navy and Army services, or under the Metropolitan Board of Works. The remarkable increase in the number of school nurses, health visitors, and borough nurses shows that the tendency for nurses to become the employees of the State or the municipality is increasing, and this tendency is accentuated by the amalgamations taking place between County Councils and the voluntary nursing associations. The Ministry of Health cannot carry out a tithe of the work which will devolve upon it without the co-operation of a contented, well trained, and zealous body of nurses, welded together, protected and recognised as a branch of the public service by a well-planned measure of State registration.

There is one other reason why Government should undertake this matter. Registration cannot be compulsory. Parliament will never consent to put impediments in the way of women rendering service to the sick as and when they may desire to do so. Consent to the State registration of nurses will not cover the imposition of penalties on nurses who do not submit their names to the registrar. It is therefore of the utmost importance that there should be no party left outside opposing registration and pandering to that weak side of human nature which ever seeks excuses for holding aloof from a movement demanding the exercise of a little trouble, the expenditure of a little money. If the State registration of nurses remain a matter of dispute between two parties in the nursing world, it is hardly possible that a movement against its acceptance will not be set afoot. We may say that nurses who neglect to get registered will do so at their own peril, but the fact remains that in so doing they would imperil the unanimity which must be attained if registration is to be a real protection to nurses and to the public. The fact that a Government measure would command general assent is the strongest point in its favour.

